# Glucocorticoid-Induced Tumour Necrosis Factor Receptor-Related Protein: A Key Marker of Functional Regulatory T Cells

**DOI:** 10.1155/2015/171520

**Published:** 2015-04-15

**Authors:** Simona Ronchetti, Erika Ricci, Maria Grazia Petrillo, Luigi Cari, Graziella Migliorati, Giuseppe Nocentini, Carlo Riccardi

**Affiliations:** Section of Pharmacology, Department of Medicine, University of Perugia, Piazza Severi 1, 06132 Perugia, Italy

## Abstract

Glucocorticoid-induced tumour necrosis factor receptor-related protein (GITR, TNFRSF18, and CD357) is expressed at high levels in activated T cells and regulatory T cells (Tregs). In this review, we present data from mouse and human studies suggesting that GITR is a crucial player in the differentiation of thymic Tregs (tTregs), and expansion of both tTregs and peripheral Tregs (pTregs). The role of GITR in Treg expansion is confirmed by the association of GITR expression with markers of memory T cells. In this context, it is not surprising that GITR appears to be a marker of active Tregs, as suggested by the association of GITR expression with other markers of Treg activation or cytokines with suppressive activity (e.g., IL-10 and TGF-*β*), the presence of GITR^+^ cells in tissues where Tregs are active (e.g., solid tumours), or functional studies on Tregs. Furthermore, some Treg subsets including Tr1 cells express either low or no classical Treg markers (e.g., FoxP3 and CD25) and do express GITR. Therefore, when evaluating changes in the number of Tregs in human diseases, GITR expression must be evaluated. Moreover, GITR should be considered as a marker for isolating Tregs.

## 1. Introduction

Regulatory T cells (Tregs) are specialized cells that control immune responses to pathogens and mediate immunological self-tolerance and homeostasis, as initially described by Sakaguchi et al. [1]. Multiple subsets of Treg populations have been described in the literature.

Naturally occurring Tregs are derived from the thymus [2, 3]. Thymus-derived Tregs (tTregs) are characterized by the expression of the transcription factor forkhead box P3 (FoxP3) and the interleukin- (IL-) 2 receptor *α*-chain CD25 [4]. Most mouse tTregs also express glucocorticoid-induced tumour necrosis factor (TNF) receptor-related protein (GITR, TNFRSF18, CD357) and OX40 (TNFRSF14, CD134), both of which are members of the TNF receptor superfamily [5]. Although tTregs are considered to be naïve T cells, several human tTregs are derived from expanded populations of tTregs and are CD45R0^+^ (e.g., memory T cells) [6]. tTregs are anergic in vitro (e.g., do not proliferate in response to TCR triggering) but appear to be a proliferating population in vivo.

Identification of tTregs is more complex in humans than in mice, because FoxP3 is transiently expressed even by activated T cells. Further, FoxP3 immunostaining requires permeabilization of cells, preventing functional experiments. CD25 is a useful surrogate marker for Tregs. However, CD25 is highly expressed in activated T cells [7–10]. The notion that tTregs exhibit higher expression levels of CD25 (CD25^high^ or CD25^bright^) is theoretically interesting but difficult to demonstrate in a standardized manner due to differences in the affinities of anti-CD25 antibodies (Abs) and flow cytometers. Furthermore, immunohistochemistry cannot discriminate between CD25^+^ and CD25^bright^ cells. In addition, some Tregs are not CD25^+^FoxP3^+^ [11]. Thus, additional markers have been examined with CD25. For example, tTregs have been identified based on CD4^+^CD25^+^CD127^−^, CD4^+^CD25^+^CD49d^−^CD127^−^, CD4^+^CD25^int^CD45RA^+^, or CD4^+^CD25^high^CD45RA^−^ status [12–14]. Despite some evidence of plasticity, tTreg phenotype and function remain fairly stable [15].

Peripherally derived Tregs (pTregs) form a more heterogeneous Treg subset and are derived from CD4^+^ effector T cells that become activated in response to microenvironmental signals [4]. Transforming growth factor- (TGF-) *β*-producing Th3 cells and IL-10-producing Tr1 cells are examples of pTreg cells [2, 3, 16]. Several studies demonstrate that some pTreg subsets express low to undetectable levels of FoxP3 and/or CD25. For example, Bacchetta et al. showed that a peripheral CD4^+^ T cell subset expressed FoxP3 and low levels of CD25 while having regulatory activity [17]. pTregs exhibit a high degree of plasticity and can expand in response to specific needs [18, 19].

Although *αβ* CD4^+^ Tregs are the best characterized Tregs, many immune cells demonstrate regulatory functions. Among the T lymphocytes, there are CD8^+^ Tregs, CD3^+^CD4^−^CD8^−^ double-negative Tregs, and *γδ* CD4^+^ Tregs.

Tregs are characterized by the expression of specific surface markers, some of which mediate immune suppression (Table 1). Tregs produce factors, such as IL-10, IL-35, granzyme B, and TGF-*β*, which also mediate immune suppression (Table 1). Suppression of effector T cells by tTregs occurs via cell-to-cell contact, whereas soluble factors mediate T cell suppression by pTregs. However, Tregs are highly complex cells. For example, CTLA-4, one of the molecules involved in cell-to-cell inhibition, is expressed by several pTregs. In addition, differences exist between the expression of Treg markers and mechanisms of regulation by mouse and human Tregs.

Expression levels of Treg markers differ among Treg subsets; many markers are not exclusively expressed in Tregs and are also markers of activated effector T cells. This complexity and the differences between human and mouse Tregs make it difficult to evaluate the roles of Tregs in human diseases and can yield contrasting results. The best molecular marker(s) to study active Tregs in autoimmune diseases and tumours remain unclear. In this review, we present data suggesting that GITR is a crucial marker of Tregs, especially functional Tregs, and can be used as a marker to purify and evaluate the number of active Tregs.

## 2. Expression of GITR in T Cells

Schaer et al., Clouthier and Watts, and our group suggested that GITR is expressed at intermediate levels in murine and human CD4^+^CD25^−^ naïve effector T cells [20–22]; similar levels were found in CD8^+^ cells. However, reinterpretation of published data suggests that this is not completely true.

After murine GITR was cloned, our group used a goat IgG polyclonal anti-GITR antibody (AF524, R&D System) to evaluate GITR expression in murine T cells [23, 24]. The antibody was specific for GITR, as demonstrated by the absence of staining in GITR knockout cells. All CD4^+^ cells expressed GITR. Similar results were obtained by another group with a rat IgG2a monoclonal anti-GITR antibody (DTA-1) [25, 26]. Conversely, Uraushihara et al., assessing GITR expression on murine CD4^+^ T with the DTA-1 antibody, found that most CD4^+^CD25^−^ T cells did not express GITR; however, a small percentage of GITR-expressing cells were found in a CD25^−^ subpopulation [27]. The basis for the contradictory results remains unclear but may be due to preincubation of cells with Fc*γ*R-blocking antibody, although theoretically this should not affect T cell staining. However, the latter study may be more reliable, as the results were similar to what is observed in humans and mice. Thus, a small population of CD4^+^CD25^−^ cells appears to express GITR at levels that are sufficient to facilitate isolation of this specific cellular subset [27, 28].

Our group obtained human data consistent with those described by Uraushihara et al. Among populations of human peripheral CD4^+^CD25^−^ cells, GITR^+^ cells possess regulatory activity and exhibit a phenotype similar to memory and Treg cells [10, 29]. Consistent with our results, a previous study reported a small subpopulation of CD4^+^CD25^−^GITR^+^ cells among human peripheral blood lymphocytes [30]. Therefore, it is reasonable to conclude that GITR is expressed in such a small population of CD4^+^ effector T cells and that depletion of GITR^+^ cells will not eliminate most CD4^+^ cells [27–29]. Indeed, Snell and colleagues have described distinct subsets of GITR^low^ and GITR^high^ cells [31]. Furthermore, real-time PCR experiments in sorted human subpopulations demonstrated that CD4^+^CD25^−^GITR^−^ cells express* GITR* mRNA at levels that are 10-fold lower than those in CD4^+^CD25^−^GITR^+^ cells [29]. Therefore, we propose that naïve CD4^+^ cells are GITR^−/low^ cells even if some antibodies at some experimental conditions suggest that most CD4^+^ and CD8^+^ cells are GITR^+^.

CD4^+^ T cells express high levels of GITR following activation. Studies suggest that GITR upregulation occurs rapidly following CD4^+^ T cell activation and peaks after one day to three days [25, 32, 33]. However, GITR does not appear to be a marker of long-term activation [10, 34]. CD8^+^ T cells express high levels of GITR following activation too [20]. As demonstrated for the first time by Shimizu et al. and McHugh et al., GITR is expressed at high levels and provides regulatory functions in peripheral and thymic CD4^+^CD25^+^CD8^−^ Tregs [26, 35] and several other Treg subsets, as discussed below.

## 3. GITR Participates in Costimulation of Effector T Cells

**Figure 1:**
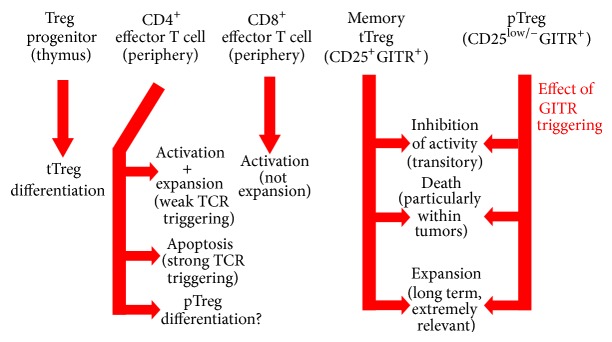
Role of GITR in CD4^+^ and CD8^+^ T cells and Tregs (thymus-derived Tregs, tTregs, and peripherally derived Tregs, pTregs) resulting from studies on rodents and humans.

GITR is triggered by the ligand GITRL, which is mainly expressed in antigen-presenting cells (APCs) and endothelial cells [36–38]. GITR is also activated by a newly described GITR ligand called SECTM1A [39]. GITR costimulation activates T cell receptor- (TCR-) triggered CD4^+^ and CD8^+^ T cells, promoting proliferation (Figure 1) [24, 25, 40–42]. GITR activation can be obtained by agonist anti-GITR Abs, soluble GITRL, or transfection of GITRL [24, 25, 40, 41, 43]. The costimulatory effect of GITR activation in T cells increases T cell expansion and cytokine production [24, 25, 40, 42], exacerbates autoimmune/inflammatory diseases [44–46], favours tumour rejection, performs viral and parasite clearance, and potentiates immune/inflammatory responses [21, 22, 47–52]. A peculiar effect of GITR costimulation is increased IL-10 production, such that neutralizing anti-IL-10 antibodies increase CD4^+^ proliferation following GITR activation [25].

GITR may have a role in CD8^+^ T cells different from CD4^+^ T cells, as initially suggested by the observation that GITR triggering exerts a different effect in alloreactive CD4^+^ and CD8^+^ T cells in GvHD [101]. One difference refers to the reciprocal interaction between GITR and CD28. During activation of CD4^+^CD25^−^ cells, GITR upregulation depends on CD28 stimulation [41, 102]. On the contrary, CD8^+^ cells cannot be stimulated by CD28 in the absence of GITR if suboptimal doses of anti-CD3 Ab are used; however, GITR can coactivate downstream functions in the absence of CD28 [103, 104]. Thus, in CD8^+^ cells, GITR is necessary for CD28 costimulatory activity. Expression of 4-1BB also depends on GITR expression in CD8^+^ memory T cells [105] and GITR promotes survival of memory bone marrow CD8^+^ T cells [106]. A specific role for GITR activation in the stimulation of CD8^+^ T cells is well-defined during chronic viral infection [34, 104, 107].

Interestingly, the number of CD8^+^ T cells is not affected when GITR is activated by a supraphysiological level of ligand in GITRL-transgenic mice [108, 109]; thus, physiological GITR activation is sufficient to fully stimulate CD8^+^ T cells. Conversely, the number and phenotype of CD4^+^ T cells are dramatically altered in two different transgenic mice that constitutively express GITRL in B cells [108] or in most APCs (i.e., majority of B cells, DCs, NK cells, and a fraction of macrophages) [109]. The most impressive phenotypic change is CD4^+^ Treg expansion, as discussed in Section 5. However, CD4^+^ effector T cell expansion and maturation are favoured as well. The number of CD4^+^ T cells with an effector memory-like (CD44^+^CD62L^−^) and central memory-like (CD44^+^CD62L^+^) phenotype increased by twofold in GITRL-B-cell transgenic mice compared to that of wild-type control mice. Robust activation of GITR in GITRL-APC-transgenic mice resulted in 10-fold activation of CD44^+^CD62L^−^ compared to that in wild-type control (at 10 weeks). Conversely, CD4^+^ naïve T cells (CD44^−^CD62L^+^) decreased by two- to threefold in transgenic mice, suggesting that GITR-triggered naïve T cells tend to be more reactive against antigens and differentiate towards the memory phenotype.

## 4. GITR Is a Crucial Player in tTreg Differentiation

Thymic development of Tregs is a two-step process [110, 111]. First, TCR and CD28 signalling induce IL-2 and chromatin remodelling at the FoxP3 locus (Treg progenitors). The second signalling event is cytokine-dependent and leads to FoxP3 expression. Mahmud et al. recently demonstrated that this step involves three TNFRSF members (GITR, OX40, and TNFR2) (Figure 1) [112]. In fact, Treg progenitors express high levels of GITR, OX40, and TNFR2. Combined neutralization of their ligands (GITRL, OX40L, and TNF) abrogates the development of Tregs and costimulation of GITR and OX40 results in a tenfold increased sensitivity to low doses of IL-2. Treg progenitors with TCRs of the highest affinity and the highest expression of GITR, OX40, and TNFR2 compete more effectively for the respective ligands and are more likely to differentiate into mature Tregs. As a consequence, GITR expression is high in mature tTregs.

## 5. GITR Is a Crucial Player in Treg Expansion

The role of GITR activation in the expansion of Tregs is supported by studies in mice (Figure 1). The number of Tregs is lower in GITR-knockout mice [23, 41, 113]. GITR activation by an anti-GITR Ab costimulates Tregs and promotes loss of anergy in the absence of IL-2 [24, 25]. In the presence of IL-2 and anti-CD3 Ab, GITR activation by a GITRL-Fc fusion protein promotes proliferation of both effector and Treg cells; however, FoxP3^+^ cells exhibit a stronger response to ligation of GITR compared to that in FoxP3^−^ cells [114]. Indeed, treatment of mice with GITRL-Fc favors a preferential proliferation of FoxP3^+^ cells [114].

Other in vivo models confirm the in vitro findings. As expected, the number of FoxP3^+^ Tregs is higher in transgenic mice that overexpress GITRL in B cells or APCs, compared to that of wild-type mice [108, 109]. Tregs from GITRL-transgenic mice are phenotypically activated and retain suppressive abilities. The costimulatory effect towards Tregs is stronger than that towards effector T cells.

Tr1 cells, which are CD4^+^ pTregs derived from activated effector T cells in peculiar conditions, do not express FoxP3 but do produce IL-10 [115]. In 12-week-old wild-type mice, 1% of splenic CD4^+^ T cells are FoxP3^−^IL-10^+^. In transgenic mice with APCs that constitutively express GITRL, the FoxP3^−^IL-10^+^ subset is 6%-7% of all CD4^+^ T cells. These findings suggest that chronic GITR/GITRL signalling favours not only expansion of FoxP3^+^ tTregs but also the expansion and/or generation of FoxP3^−^ Tr1-like cells [109]. Suppression by these Tr1-like cells is mediated by IL-10, as indicated by experiments using a neutralizing anti-IL-10 monoclonal antibody. The Tr1-like cells in transgenic mice are GITR^+^, suggesting that GITR is a marker of murine Tr1 cells and participates in their expansion. Interestingly, GITR activation of CD4^+^ effector T cells stimulates IL-10 production [25]. Generation of in vitro induced Tregs (iTregs) is induced in cocultures of CD4^+^ effector T cells and malignant plasma cells, and iTregs show high levels of GITR expression [74]. However, the effects of blocking GITR have not been tested.

Studies on GITRL^−/−^ mice confirm the role of GITR activation in Treg expansion and/or generation [116]. In fact, the expansion or generation of Tregs is impaired in GITRL^−/−^ mice after injection of the dendritic cell-inducing factor Flt3 ligand. Furthermore, the expansion or generation of OVA-specific Tregs is impaired after gene transfer of ovalbumin (using the adenoassociated virus AAV8-OVA). Considering that GITR activation is elicited even by the newly discovered GITR ligand, SECTM1A [39], it is possible that these results would be even more relevant if GITR activation was abolished by loss of both GITR ligands (GITRL and SECTM1A).

In multiple sclerosis, treatment with IFN-*β* increases the number of CD4^+^CD25^+^FoxP3^+^ Tregs following increased expression of GITRL in CD14^+^ monocytes [61]; this may suggest that GITR activation favours Treg expansion in humans too. However, so far no study demonstrates the role of GITR in human Treg expansion.

## 6. Pharmacological Activation of GITR Transiently Inhibits Treg Activity

GITR stimulation enhances T cell proliferation/activation not only through costimulation of effector T cells but also by the inhibition of Tregs (Figure 1), as originally demonstrated by the Sakaguchi and Shevach groups using anti-GITR Abs [26, 35]. Other studies have demonstrated the same effect when GITR was activated by GITRL overexpressed in APCs [24, 40, 117, 118]. GITR stimulation abolishes the activity of other suppressor cells, such as retinal pigment epithelial cells [119] or CD4^+^CD25^−^ T cells in aged mice [120]. The same effect is elicited when human Tregs are exposed to anti-GITR Abs [29, 121].

Some studies indicate that increased proliferation of effector cells in response to GITR activation of effector T cells renders them more resistant to Treg suppression [122]. In this context, two studies suggest that GITR stimulation activates an unknown pathway in effector T cells distinct from that which is activated by CD28, blocking immunosuppression [41, 119]. However, other studies demonstrate that GITR stimulation activates transduction pathways in Treg cells that are responsible for Treg suppression. In particular, GITR signalling downregulates granzyme B [123], degrades FoxP3 protein [124, 125], phosphorylates c-Jun N-terminal kinase (JNK), and activates NF-*κ*B [126]. Furthermore, in vitro and in vivo experiments demonstrate that stimulation of GITR on Tregs underlies the increased activation of effector T cells during inflammatory responses or tumour rejection [24, 26, 45, 124–127].

The full activity of Tregs in GITRL-transgenic mice [108, 109] suggests that inhibition of Treg suppression is transient and may be due to overstimulation of GITR in a nonphysiological condition (pharmacological effects). Indeed, GITR-dependent decrease in FoxP3 protein expression is not due to changes in the levels of* FoxP3* mRNA, confirming that this is a transient effect.

Some studies demonstrate that the effect of anti-GITR Ab on the suppressive activity of Tregs depends on Treg depletion [128, 129], which in turn depends on the binding of anti-GITR Ab with activating Fc *γ* receptors (Fc*γ*R) [129]. The increased susceptibility of Tregs compared to that of activated CD4^+^ and CD8^+^ cells may be due to higher levels of GITR expression on Tregs. Studies suggest that the effect of anti-GITR Ab is tumour-specific and may depend on myeloid cells and natural killer cells present in tumours but not in draining lymph nodes [129].

In conclusion, GITR activation has four distinct effects on Treg/effector cell interplay: (1) transient inhibition of Treg regulatory activity, (2) decreased sensitivity of effector T cells to Treg suppression, (3) killing of Tregs (at least within solid tumours), and (4) increased proliferation and expansion of the Treg compartment.

The most relevant of these four remains a matter of debate. The relative importance of each of these mechanisms depends on the context and the disease [21, 130]. Nonetheless, the balanced effects on Tregs may reduce the chances of adverse effects. Indeed, no overt autoimmunity was observed in adult animals treated with anti-GITR Abs [131, 132].

## 7. GITR Is a Marker of Murine Tregs

The evidence supporting a crucial role for GITR in the maturation of tTregs and expansion of tTregs and pTregs includes the fact that tTregs and expanded pTregs constitutively express GITR. Indeed, GITR is expressed in many (if not all) subsets of Tregs.

Constitutive expression of GITR in murine tTregs was first described by McHugh et al. [35] and Shimizu et al. [26] and was confirmed by Zelenika et al. [133]. Cells with suppressive activity express high levels of GITR in FoxP3 transgenic mice [134]. Indeed, gene expression in the* Gitr* locus is regulated by NF-*κ*B and FoxP3 through an enhancer [135]. In FoxP3 transgenic mice, GITR expression is observed surprisingly in CD25^−^ cells, demonstrating that GITR represents a Treg marker independent of CD25 [134]. Uraushihara et al. demonstrated that cotransfer of the CD4^+^GITR^+^ population prevents the development of CD4^+^CD45RB^high^ T cell-transferred colitis [27]. Interestingly, CD4^+^GITR^+^ T cells prevent wasting disease and colitis independently of CD25 expression. In fact, both CD4^+^CD25^+^GITR^+^ and CD4^+^CD25^−^GITR^+^ T cells express CTLA-4, show anergy, suppress T cell proliferation, and produce IL-10 and TGF*β*. In GITRL-transgenic mice, the expanded CD4^+^FoxP3^−^IL-10^+^ Tr1-like cells are GITR^+^ [109], suggesting that GITR expression in Tregs is FoxP3-independent.

In summary, several subsets of murine Tregs appear to express GITR. GITR expression was observed in *αβ* CD4^+^CD25^+^ Tregs [20, 36, 38] and some CD4^+^ Treg subsets (e.g., CD25^+^CD4^+^CD103^+^ cells [136] and CD25^+^CD4^+^CD83^+^ cells [137]), which are collectively called tTregs. GITR is expressed by Tr1 Tregs too, which are defined by their capacity to produce high levels of IL-10 and lack of FoxP3 [109, 138]. Moreover, GITR is expressed in CD8^+^CD25^+^ Tregs [139–141], *γδ* CD25^+^ Tregs [142], and TCR^+^CD4^−^CD8^−^CD25^+/−^PD-1^high^FoxP3^−^ cells (double-negative Tregs) [143].

Functional studies confirm that GITR is a highly specific marker for identifying and isolating Tregs. Ono et al., for example, demonstrated that the transfer of GITR^+^-depleted T cell populations caused death in 90% of nude mice due to autoimmune diseases [28]. In contrast, injection of CD25^+^-depleted cells did not cause death. The same study demonstrates that the transfer of GITR^+^-depleted T cells from NOD mice to NOD-SCID mice promotes the accelerated development of diabetes as compared to those transferred with CD25^+^-depleted T cells (one month after cell transfer versus more than two months). The former mice died or had to be sacrificed because of debilitation before or at seven weeks after transfer, whereas only 20% of the latter (transferred with CD25-depleted T cells) died at 12 weeks after transfer.

These studies demonstrate that GITR is expressed in many (if not all) Treg subsets and at much lower levels in effector T cells. Because it also allows CD25^−^ Tregs to be isolated, GITR appears to be a more useful marker than CD25. The big difference among the diseases observed in GITR^+^ cell-depleted and CD25^+^ cell-depleted mice suggests that either known CD25^−^ Tregs (e.g., Tr1 and double-negative cells) are crucial to immune homeostasis or the number of CD25^−^GITR^+^ cells is big and other CD25^−^GITR^+^ cell subsets with regulatory activities exist in mice.

## 8. GITR Is a Marker of Human Tregs

Soon after the discovery of GITR as a marker of murine Tregs [26, 35], studies reported that GITR is coexpressed with CD25 and FoxP3 in human CD4^+^ tTregs [121, 144, 145] and some of their subsets (e.g., FoxP3^+^Tim^+^ [83]). CD4^+^CD25^low/−^GITR^+^ cells isolated from healthy donors express FoxP3 and show regulatory activity [29]. These cells are likely to be pTregs, because they express CD45RO, IL-10, and TGF-*β*. Thus, CD4^+^CD25^low/−^GITR^+^ cells are similar to human Tr1 clones [146] and FoxP3^−^IL-10-producing Tr1-like cells that have expanded in GITRL-transgenic mice [109]. Finally, GITR is found also in CD8^+^CD25^+^ and CD8^+^FoxP3^+^ human Tregs [70, 90, 147, 148].

Therefore, GITR is used as a human Treg marker in several studies. A PubMed search revealed 49 studies in the past three years that investigated the roles of Tregs in autoimmune/allergic diseases, tumours, and infections and reported GITR as a Treg marker (Table 2). More interestingly, GITR is found in memory/activated Tregs, as reported below.

## 9. GITR Is Frequently Found in Memory and/or Active Tregs

Most Tregs, including tTregs, are memory Tregs and GITR expression occurs more frequently in these cells. Uraushihara et al. assessed correlations among GITR, CD25, and CD45RB expression in freshly isolated murine splenic CD4^+^ T cells [27]. Both CD25^+^ and CD25^−^ Tregs expressed GITR; GITR was exclusively expressed on CD4^+^CD45RB^low^ cells, suggesting that CD4^+^GITR^+^ cells are memory cells. We recently described a human CD4^+^CD25^low/−^GITR^+^ Treg subset [10, 29, 58]. The majority of these cells are CD45RA^−^ and CD45RO^+^, demonstrating that CD4^+^CD25^low/−^GITR^+^ cells are memory Tregs and most likely pTregs. The association of GITR with a memory phenotype is not surprising considering that GITR participates in Treg expansion. Indeed, an expanded population of CD4^+^CD25^low/−^GITR^+^ Tregs is found in some patients with Sjogren's syndrome and SLE [10, 58]; expansion is observed in patients with inactive disease, suggesting that CD4^+^CD25^low/−^GITR^+^ Tregs participate in disease control.

Several studies suggest that GITR is a marker of Tregs that are actively suppressing effector cells. This is supported by the association of GITR expression with other Treg activation markers (e.g., CTLA-4), the expression of GITR in antigen-specific Tregs and/or T cells that produce cytokines with suppressive activity (e.g., IL-10 and TGF-*β*), the presence of GITR^+^ cells in tissues with active Tregs (e.g., solid tumours), and functional studies on Tregs. Our purpose is to illustrate the relevance of GITR as a marker of human Tregs; thus, we focus mainly on studies performed in humans.

Wang et al. characterized CD4^+^ and CD8^+^ T cell clones derived from the peripheral blood of patients infected with human herpes virus 6 (HHV-6) [90]. They found that HHV-6-specific T cell clones with suppressive activities expressed not only CD25 and FoxP3, as expected, but also GITR; furthermore, nonsuppressive HHV-6-specific CD4^+^ or CD8^+^ T cells were negative for all three markers. Similar data were found in CD8^+^ Tregs from mice, in which FoxP3^+^ Tregs expressed CD25, GITR, and IL-10 [140]. Consistent with the Wang study, our group recently observed elevated expression of FoxP3, TGF-*β*, and CTLA-4 in CD4^+^CD25^+^GITR^+^ PBMCs compared to that of CD4^+^CD25^+^GITR^−^ PBMCs (unpublished data). In human decidua, expression of GITR is higher in CD25^+^CTLA-4^+^ cells compared to that in CD25^+^CTLA-4^−^ [149] and suppressive activity correlates with the intensity of GITR and CTLA-4 expression in human CD4^+^CD25^+^ clones [150]. Similar findings have been described in human Tr1 clones (GITR^+^CD25^low^), in which FoxP3 downregulation results in the loss of GITR surface expression and Treg suppressive activity but does not alter CD25 expression [151].

Treg dysregulation (decreased number or function) has been demonstrated in several autoimmune diseases. GITR expression appears to be lower in patients with type 1 diabetes (T1D) compared to that of controls, according to two studies. In the first study, the percentages of CD4^+^CD25^high^CD127^dim⁡/−^ cells did not differ between children with T1D and controls, but mRNA levels of several Treg markers, including GITR, were lower in Tregs from children with T1D [152]. In the second study, the number of CD25^low^ cells, especially CD4^+^CD25^low/−^GITR^+^ cells, was lower in T1D patients than in controls [60]. Even within the CD4^+^CD25^high^ population, the number of GITR^+^ cells was lower in T1D patients, indicating that GITR^+^ Tregs (independent of FoxP3 or CD25 expression) are less abundant in T1D patients. These data suggest that GITR^+^ Tregs are crucial for the control of T1D.

In several solid tumour types, a subset of tumour-infiltrating lymphocytes is formed by antigen-specific Tregs that inhibit other immune cells and prevent tumour rejection. Data from animal tumour models suggest that GITR^+^ Tregs tend to infiltrate tumours. For example, Sacchetti et al. demonstrated that GITR expression in Tregs (CD4^+^FoxP3^+^ cells) residing in a B16 melanoma is approximately 10-fold higher than that of Tregs in the spleens of the same animals [153]. In Colon26 tumours, Tregs found in tumour infiltrating lymphocytes (TILs) show approximately fourfold stronger GITR signals than those of the equivalent lymph node population [129]. The role of GITR as a TIL Treg marker has also been confirmed in humans. In head and neck squamous cell carcinoma (HNSCC), CD25^high^ cells were enriched and represented 3% of CD3^+^CD4^+^ TILs compared to circulating CD3^+^CD4^+^ T cells of the same patients, which comprised 1% of CD3^+^CD4^+^ cells; circulating CD3^+^CD4^+^ T cells of normal controls comprised 0.4% of CD3^+^CD4^+^ cells [154]. Analysis of markers expressed on CD25^high^ cells among PBMCs and TILs from the same patients shows that some markers (e.g., FoxP3 and CTLA-4) are found in both Tregs at the same levels but others are not. In particular, a lower percentage of TIL Tregs express CCR7 (77% in TIL Tregs versus 49% in PBMC Tregs) and CD62L (28% versus 47%). A higher percentage of TIL Tregs express CD132 (20% versus 40%). TIL Tregs are fully active, as suggested by expression of FasL (57% versus 0%), intracellular IL-10 (71% versus 0%), and intracellular TGF*β* (97% versus 0%) and as demonstrated by a much higher suppressive activity compared to that of PBMC Tregs from the same patients. Importantly, IL-10/TGF*β*-producing TIL Tregs are best detected by GITR, which is expressed in 83% of TIL Tregs and about 5% of PBMC Tregs of patients. Thus, GITR is the main marker of active Tregs, at least in HNSCC tumour. Similar findings were found in glioblastoma tumours, where all tTregs (CD4^+^FoxP3^+^Helios^+^) were GITR^+^ [6].

A study on patients with invasive breast cancer confirms the role of GITR, even if data were less impressive [78]. FoxP3 and CTLA-4 are expressed in about 20% of CD25^high^ cells among PBMCs and about 30% of the CD25^+^ cells infiltrating the tumours, resulting in an increase of about 1.5-fold in TILs. In contrast, GITR is expressed in about 5% of the CD25^high^ cells of PBMCs and about 30% of the CD25^+^ cells infiltrating the tumours, resulting an increase of about sixfold in TILs. In PBMCs of the patients, the mean frequency of CD25^high^ and CD25^low^ cells was more than twofold higher than in healthy controls and expression of GITR, CTLA-4, and CCR4 was significantly higher in both subsets [78]. Comparing T-lymphocyte phenotypes among the lymph nodes of three breast cancer patients, Krausz et al. demonstrated that CD4^+^CD25^low^CD127^+^GITR^+^ cells are more abundant in tumour-positive lymph nodes than in tumour-negative lymph nodes of the same patient [77]. These findings suggest that tumour cells stimulate expansion of this Treg subset or that GITR^+^ cells tend to accumulate within the tumour.

Studies on other tumours confirm the overall picture. Padovani et al. studied cervical tissue of healthy women in comparison with tissue from women infected with papillomavirus (HPV) or women with carcinoma [84]. Most (85%) of carcinoma samples showed high GITR expression, whereas only 41% of normal and low-grade cervical intraepithelial neoplasia samples showed high GITR expression. Moreover, most (78%) of the samples highly positive for HPV showed high GITR expression, whereas only 43% of the samples negative for HPV showed high GITR expression. No differences in CD25 staining were observed, suggesting that the presence of GITR^+^ Tregs correlates with malignancy and HPV infection of the cervical stroma and possibly with Treg activity. von Rahden et al. demonstrated that GITR expression in TILs is associated with oesophageal adenocarcinomas in the absence of Barrett's mucosa [155]. In patients with hepatocellular carcinoma and liver metastases from colon cancer, the number of CD25^+^FoxP3^+^ Tregs is significantly higher in tumours than that in tumour-free liver tissue. The MFI of GITR and ICOS are significantly higher in Tregs from tumours than in those from tumour-free liver tissue [81].

In conclusion, GITR is associated with activation and increased suppressive activity of Tregs in both mice and humans.

## 10. Conclusions

GITR is a crucial player in differentiation of tTreg and expansion of Tregs, including both tTregs and pTregs. Indeed, some data suggest that GITR is associated with markers of memory cells. Approximately 70–80% of Tregs are memory T cells, and expanded Tregs are active, at least in some diseases (e.g., several solid tumour types); thus, it is not surprising that several studies support GITR as a marker of active Tregs. Moreover, some Treg subsets, including Tr1 cells, express low levels of classical Treg markers (e.g., FoxP3 and CD25) or do not express them at all despite exhibiting expression of GITR. For this reason, we believe that GITR^+^ Tregs must be evaluated when considering increased or decreased numbers of Tregs in human diseases. Finally, the use of GITR as a marker to isolate Tregs should be considered as we have recently proposed [156].

## Figures and Tables

**Table 1:** Markers of Tregs.

	Marker^1^	Localization	Expressed by
Involved in establishing Tregs phenotype	FoxP3	Nucleus	tTregs, pTregs (but not in some subsets), and CD8^+^ Tregs
	Helios	Nucleus	tTregs, pTregs (occasionally)

Involved in Tregs regulatory activity and markers of active Tregs	IL-2 receptor (CD25)	Membrane (the level of expression is crucial)	tTregs, pTregs (but not in some subsets), and CD8^+^ Tregs
	CTLA-4	Membrane (but detectable only intracellularly)	tTregs, pTregs
	TGF-*β*	Cytoplasm, secreted	pTregs (especially but not exclusively Th3)
	IL-10	Cytoplasm, secreted	pTregs (especially Tr1)
	Granzyme B	Cytoplasm, secreted	tTregs, pTregs
	Perforin	Cytoplasm, secreted	tTregs
	IL-35	Cytoplasm, secreted	pTregs (occasionally)
	E5NT (CD73)	Membrane	pTregs
	ENTPD1 (CD39)	Membrane	pTregs
	PD-1 ligand (PD-L1)	Membrane	pTregs

Other markers of active Tregs	GITR (CD357)	Membrane	tTregs, pTregs, and CD8^+^ Tregs
	T-cell Ig and mucin domain protein-3 (Tim-3)	Membrane	tTregs, pTregs
	Galectin-9	Membrane	tTregs, pTregs
	ICOS (CD278)	Membrane	pTregs
	latency-associated peptide (LAP)	Membrane	tTregs
	CD69 (C-type lectin receptor)	Membrane	pTregs

Involved in tTregs differentiation	IL-2 receptor (CD25)	Membrane	tTregs
	GITR (CD357)	Membrane	tTregs
	OX40 (CD134)	Membrane	tTregs
	TNFR2	Membrane	tTregs

Involved in pTregs differentiation/expansion and tTregs expansion	IL-2 receptor (CD25)	Membrane	tTregs, pTregs (but not in some subsets)
	CD28	Membrane	tTregs, pTregs
	GITR (CD357)	Membrane	tTregs, pTregs, and CD8^+^ Tregs
	OX40 (CD134)	Membrane	tTregs, pTregs
	ICOS (CD278)	Membrane	pTregs
	LAG-3 (CD223)	Membrane	tTregs, pTregs
	Programmed cell death (PD)-1	Membrane	pTregs
	PD-1 ligand (PD-L1)	Membrane	pTregs
	CD226	Membrane	pTregs (Tr1)
	CD69 (C-type lectin receptor)	Membrane	pTregs

Involved in inhibition of Tregs activity	GITR (CD357)	Membrane	tTregs, pTregs, and CD8^+^ Tregs
	OX40 (CD134)	Membrane	tTregs, pTregs
	4-1BB (CD137)	Membrane	tTregs, pTregs

Other markers	CD45R0 (memory marker)	Membrane	tTregs (those expanded), pTregs
	CD45RA (naïve marker)	Membrane	tTregs
	Neuropilin-1 (VEGF receptor)	Membrane	tTregs, low expression in pTregs
	CD49b	Membrane	pTregs (Tr1)

Not expressed by Tregs	IL-7 receptor (CD127)	Membrane	tTregs
	CD49d	Membrane	tTregs

^1^Expression is not exclusive of Tregs.

**Table 2:** Original studies considering GITR as a Treg marker in humans. Studies published in the last three years (2012–2014) were identified from a PubMed database search.

Disease area	Specific disease	Treg phenotype	Comment	Reference
Autoimmune/allergic diseases	Rheumatic diseases	CD4^+^CD25^+^	Increased GITR expression in CD4^+^CD25^+^ cells from peripheral blood of patients with severe rheumatoid arthritis	[53]
		CD4^+^CD25^+/high^CD127^low/−^	About 35% of Tregs is GITR^+^ in peripheral blood of rheumatoid arthritis and osteoarthritis patients; in the same patients, about 46% of Tregs is GITR^+^ in synovial membranes	[54]
		CD4^+^CD25^low/−^GITR^+^	CD4^+^CD25^low/−^GITR^+^ cell population is expanded in the peripheral blood of SLE patients with inactive disease	[10]
		CD4^+^CD25^+^, CD4^+^CD25^high^ and CD4^+^CD25^+^CD127^low/−^	The expression of GITR in Tregs positively correlates with SLE disease activity index (SLEDAI) in SLE patients	[55]
		CD25^+/high^CD127^−/low^FoxP3^+^	Decrease in the GITR^+^ Treg/GITR^+^ effector T cell ratio in SLE^1^ patients	[56]
		CD4^+^CD25^−^GITR^high^	CD4^+^CD25^−^GITR^high^ cell population is expanded in the peripheral blood of Sjogren's syndrome patients	[57]
		CD4^+^CD25^low^GITR^+^	CD4^+^CD25^low/−^GITR^+^ cell population is expanded in the peripheral blood of Sjogren's syndrome patients with inactive disease	[58]
		Not applicable	Presence of GITR^+^ cells in lymphocytic foci and periductal areas of the labial salivary glands of Sjogren's syndrome patients	[59]
	Diabetes	CD4^+^CD25^high^	A significant decrease of GITR^+^ cells, GITR mean fluorescence intensity, and GITR mRNA expression within the Treg population was observed in type 1 diabetes patients compared with healthy controls	[60]
	Multiple sclerosis	CD4^+^FoxP3^+^	In multiple sclerosis patients treatment with INF*β* increases Treg number as a consequence of GITRL expression in monocytes	[61]
	Atopic diseases	Not applicable	The expression of FoxP3, GITR, and LAG3 was used to assess the effect of IL-10 polymorphisms on Treg number	[62]
		Not applicable	The expression of FoxP3, GITR, and LAG3 was used to assess the effect of STAT6 polymorphisms on Treg number	[63]
	Asthma	CD4^+^CD25^high^CD127^low^	The expression of GITR in Tregs isolated from severe allergic asthma patients was similar to that from mild to moderate asthma	[64]
	Vascular diseases	CD4^+^CD25^+^FoxP3^+^	The mRNA levels of GITR resulted significantly lower in Kawasaki disease patients as compared to healthy controls	[65]
		CD4^+^CD25^+^FoxP3^+^	Significant reduction (4-fold) in relative expression of GITR mRNA in immune thrombocytopenic purpura patients as compared to healthy controls (*P* = 0.006)	[66]
	—	Not applicable	Studying the modulation of immune system development in new born, the mRNA expression of FoxP3, GITR, and LAG3 was used to assess Treg expansion in cord blood	[67]

Transplantation		CD4^+^CD25^+^	Studying the expansion of pTreg in allogeneic culture, FoxP3, GITR, and CTLA-4 were used as markers of Treg activity	[68]
		CD4^+^CD25^high^	Studying the effect of autologous hematopoietic stem cell transplantation in multiple sclerosis patients, GITR and CTLA-4 were used as markers of Treg activity	[69]
		CD8^+^CD25^+^	Studying iCD8 T cells generated in response to allogeneic dendritic cells, GITR, CTLA-4, and FoxP3 were used as markers of Treg differentiation	[70]

Immunodeficiency		CD4^+^CD25^+^FoxP3^+^	Expression of GITR and CTLA-4 was used as markers of Treg activity	[71]

Cardiomyopathy		GITR^+^	GITR^+^ Tregs are increased in human dilated cardiomyopathy	[72]

Tumors	Leukemia and lymphoma	CD4^+^GITR^+^	CD4^+^GITR^+^ cells are increased in multiple myeloma patients	[73]
		CD4^+^CD25^+^FoxP3^+^	GITR is a marker of in vitro induced Tregs by coculture with multiple myeloma cells	[74]
		CD4^+^GITR^+^	About 50% of CD4^+^ cells infiltrating Hodgkin's lymphoma are GITR^+^	[75]
		CD4^+^CD25^+^FoxP3^+^	GITR expression is modulated in Tregs from thalidomide-treated patients	[76]
	Breast tumors	CD4^+^CD25^high^GITR^+^CD127^−/low^ and CD4^+^CD25^low^GITR^+^CD127^high^	Both Treg subsets are increased in tumor-positive lymph nodes	[77]
		CD25^+^CD4^+^	Expression of FoxP3, GITR, CTLA-4, and CD103 was tested as markers of Treg activity	[78]
	Colorectal carcinoma	CD4^+^CD25^high^FoxP3^+^GITR^+^	CD4^+^CD25^high^FoxP3^+^GITR^+^ cell subset is increased 3-fold in the PBMC of patients with colorectal carcinoma	[79]
	Brain tumor	CD4^+^CD25^bright^	GITR is one of the markers of Tregs in PBMC	[80]
	Liver cancer	CD4^+^CD25^+^FoxP3^+^	Tumor Tregs express higher levels of GITR than Tregs in tumor-free liver tissue and blood	[81]
	Ovarian cancer	Not applicable	Poorer survival was associated with the minor allele at SNPs in TNFRSF18/TNFRSF4 in patients with mucinous ovarian cancer (rs3753348, *P* = 9.0 × 10^−4^)	[82]
	Hepatocellular, cervical, colorectal, and ovarian carcinoma	Not applicable	Expression of CD25, FoxP3, CTLA-4, and GITR was higher in CD4^+^Tim-3^+^ than in CD4^+^Tim^−^ cells infiltrating tumors; moreover, most CD4^+^Tim-3^+^ cells isolated from the paired nontumor tissues and peripheral blood did not express CD25, FoxP3, CTLA-4, and GITR	[83]
	Cervical carcinoma	GITR^+^	High GITR expression was observed in both cervical carcinoma and high-grade squamous intraepithelial lesion samples	[84]

Infection	Viral	CD4^+^CD25^high^FoxP3^+^	In human immunodeficiency virus- (HIV-) infected patients with high immune activation and low-level CD4 T-cell repopulation under suppressive high active antiretroviral therapy, Tregs show enrichment in CTLA-4 and GITR markers, compared with the HIV controls and healthy subjects	[85]
		CD4^+^FoxP3^+^	The expression of CTLA-4 and GITR is decreased in T cells from PBMC of human T-lymphotropic virus-1 associated myelopathy/tropical spastic paraparesis patients as compared to healthy donors	[86]
		GITR^+^	Among all samples with high GITR expression, 77% were human papilloma virus positive; among samples negative for intraepithelial lesion and malignancy, only 33% had high GITR expression	[84]
		Not applicable	CTLA-4, GITR, CD103, CD25, CD69, IL-10, and TGF-*β*1 expression in PBMCs of hepatitis E virus infected patients were significantly elevated	[87]
		CD4^+^CD25^+^FoxP3^+^ and CD4^+^CD25^−^FoxP3^+^	Significantly higher expression of CTLA-4, PD-1, GITR, CD95, CD103, and CD73 on Tregs was detected in the hepatitis E virus infected patients as compared to healthy donors	[88]
		Not applicable	Epstein-Barr virus infected cord blood dendritic cells drive Tregs development by inducing the expression of FoxP3 and CTLA-4, decreasing the expression of GITR, and promoting the generation of intracellular IL-2 and IL-10	[89]
		Not applicable	Human herpes virus 6 (HHV-6) infection induces both CD4^+^ and CD8^+^ HHV-6-specific Tregs; these HHV-6-specific Tregs have potent suppressive activity and express high levels of CD25, FoxP3, and GITR	[90]
	Parasitic	CD25^high^GITR^+^	The frequency of CD25^high^GITR^+^ Tregs is similar in the peripheral blood of chronic dermal leishmaniasis patients and asymptomatically infected individuals	[91]
		Not applicable	The -163C/T (ss491228440) polymorphism in TNFRSF18 gene is not a susceptibility factor in uncomplicated malaria and parasitaemia in Congolese children	[92]
		FoxP3^+^	The expression of CTLA-4, GITR, LAG-3, and IL-10 was significantly higher in Treg from filarial-infected patients compared with that in healthy controls	[93]

In vitro studies		CD4^+^CD25^+^	Emodin treatment of dendritic cells increases the number of Tregs, which express lower levels of HLA-DR, GITR, and CTLA-4	[94]
		CD4^+^FoxP3^+^	All FoxP3^+^ invariant NKT cells display CD25 but not necessarily CTLA-4 or GITR	[95]
		CD25^high^CD45RA^−^	CD25^high^CD45RA^−^ in vitro induced Tregs (iTregs) express high levels of FoxP3, GITR, and CTLA-4 and low levels of CD127	[96]
		CD4^+^IFN*γ* ^+^	CD4^+^CD25^−^CD127^+^ effector T cells from human peripheral blood can convert into T cells with regulatory activity while concomitantly secreting IFN*γ*. Upon short-term culture in vitro these cells expressed a panel of common Treg markers, including FoxP3, CD25, GITR, HLA-DR, and CTLA-4	[97]
		Not applicable	FoxP3^+^ T cells were differentiated from CD4^+^CD25^−^ T cells (iFoxP3^+^ T cells); GITR and CTLA-4 resulted as the only Treg markers at higher levels in iFoxP3^+^ than in iFoxP3^−^ T cells	[98]
		CD4^+^FoxP3^+^	miR-126 silencing reduces the expression of FoxP3 on Tregs, which is accompanied by decreased expression of CTLA-4 and GITR, as well as IL-10 and TGF-*β*, and impairs its suppressive function	[99]
		CD4^+^CD25^high^FoxP3^+^	PIM1 kinase phosphorylates FoxP3 at serine 422 to negatively regulate its activity; knockdown of Pim1 in in vitro expanded human Tregs promotes FoxP3-induced target gene expression, including CD25, CTLA-4, and GITR, weakens FoxP3-suppressed IL-2 gene expression and enhances the immunosuppressive activity of Tregs	[100]

^1^CTLA-4: cytotoxic T-lymphocyte antigen 4; FoxP3: forkhead box P3; HLA-DR: human leukocyte antigen DR; IL-10: interleukin-10; IL-2: interleukin-2; LAG-3: lymphocyte-activation gene 3; PBMC: peripheral blood mononuclear cells; PD-1: programmed cell death-1; SLE: systemic lupus erythematosus; TNFRSF18: GITR; TNFRSF4: OX40 (CD134).
